# *Abiotrophia defectiva* causing infective endocarditis with brain infarction and subarachnoid hemorrhage: a case report

**DOI:** 10.3389/fmed.2023.1117474

**Published:** 2023-05-03

**Authors:** Miaojuan Yang, Yanxia Lin, Xin Peng, Jingsong Wu, Bo Hu, Yitao He, Jian Lu

**Affiliations:** ^1^Department of Neurology, The Second Clinical Medical College of Jinan University, Shenzhen, Guangdong, China; ^2^The First Affiliated Hospital of Southern University of Science and Technology, Shenzhen, Guangdong, China; ^3^Shenzhen Clinical Research Center for Geriatrics, Shenzhen People's Hospital, Shenzhen, China; ^4^Department of Infectious Diseases, Shenzhen University General Hospital, Shenzhen, China; ^5^Department of Cardiology, Huazhong University of Science and Technology Union Shenzhen Hospital, Shenzhen, Guangdong, China; ^6^Department of Laboratory Medicine, Shenzhen People's Hospital, Shenzhen, China

**Keywords:** *Abiotrophia defectiva*, infective endocarditis, brain infarction, subarachnoid hemorrhage, microbial mass spectrometry

## Abstract

**Introduction:**

A rare pathogen of Infective Endocarditis (IE), the *Abiotrophia defectiva*, has been known to trigger life-threatening complications. The case discussed here is of a teenager with brain infarction and subarachnoid hemorrhage caused by IE due to *A. defectiva*.

**Case report:**

A 15-year-old girl with movement disorders involving the left limbs and intermittent fevers was admitted to the hospital. A head CT scan revealed cerebral infarction in the right basal ganglia and subarachnoid hemorrhage. Moreover, vegetation on the mitral valve were confirmed by echocardiography. The blood cultures were found to be positive for Gram-positive streptococcus and identified by Vitek mass spectrometry as *A. defectiva*. She was prescribed vancomycin antibacterial therapy and underwent a surgical mitral valve replacement.

**Conclusion:**

This case is suggestive of the fact that *A. defectiva* is a rare but crucial pathogen of IE-associated stroke. Obtaining early blood cultures and using microbial mass spectrometry could help achieve an accurate diagnosis. Moreover, reasonable anti-infective medications and surgical interventions need to be combined to avoid and/or manage severe complications.

## Introduction

*Abiotrophia defectiva* (*A. defectiva*), was originally known to be a Nutritionally Variant Streptococci (NVS). This NVS was subsequently reclassified as *Abiotrophia defectiva, Granulicatella adiacens, Granulicatella elegans*, and *Granulicatella balaenopterae* through 16S rRNA gene sequencing. These organisms are normal colonization bacteria of the human body. *A. defectiva* needs L-cysteine, pyridoxal, and other such factors for its proper growth. In earlier case reports, the subacute IE were the most frequently reported cases of *A. defectiva* due to their indolent clinical course. Nevertheless, *A. defectiva* led to severe complications including valvular damage, congestive heart failure, and events of embolisation. Thus, early diagnosis and effective treatment strategy for *A. defectiva* become crucial in clinical practice.

## Case description

A 15-year-old girl was admitted to Shenzhen People's Hospital Neurology Intensive Care Unit (NICU) due to the sudden onset of a left-sided movement disorder. She experienced a sudden episode of weakness in her left lower limb one and a half months prior to hospitalization, which resolved spontaneously 3 days later. A month ago, she developed weakness in her left upper extremity, which disappeared after 5 days. Intermittent fevers of 39°C started 1 week ago, along with mild shortness of breath but no chills, cough, diarrhea, or bladder irritation symptoms. She noticed the sudden onset of a persistent left-limb movement disorder 3 h before being admitted to the NICU. She claimed to have lost 7.5 kilograms (17 percent of her body weight) in 3 months. She underwent orthodontic treatment a year ago and subsequently wore braces. She denied having a history of hypertension, diabetes, cigarette smoking, alcohol consumption, intravenous drug use, immunosuppressant use, atrial fibrillation, and inherited diseases.

She was conscious on physical examination, and her verbal responses to questions were appropriate. Her face was pale, her left lower limb muscle strength was grade II, her left upper limb muscle strength was grade III, and both Babinski and Brudzinski's signs were positive. In addition, a grade 4–6 systolic murmur was heard in the mitral region, splenomegaly was confirmed by abdominal palpation, and clubbed fingers were observed upon careful inspection of the hands.

Laboratory analysis showed the following: a normal leukocyte count, but mild normocytic anemia (hemoglobin, 91 g/L); an elevated C-reactive protein level of 32.05 mg/L (normal range, 0–5 mg/L); and a slightly elevated procalcitonin level (0.07 ng/mL; normal range, <0.05 ng/mL). The NT-proBNP level was 1,123 pg/m (normal range, <125 pg/mL). The cerebrospinal fluid (CSF) was red, the pressure was 180 mm H_2_O, the glucose level decreased (1.79 mmol/L; normal range, 3.9–6.1 mmol/L), the protein concentration increased (0.55 g/L; normal range, 0.15-0.45 g/L), the nucleated cell number increased (280 /μL; normal range, <20 /μL), and the CSF culture was negative.

**Figure 1:**
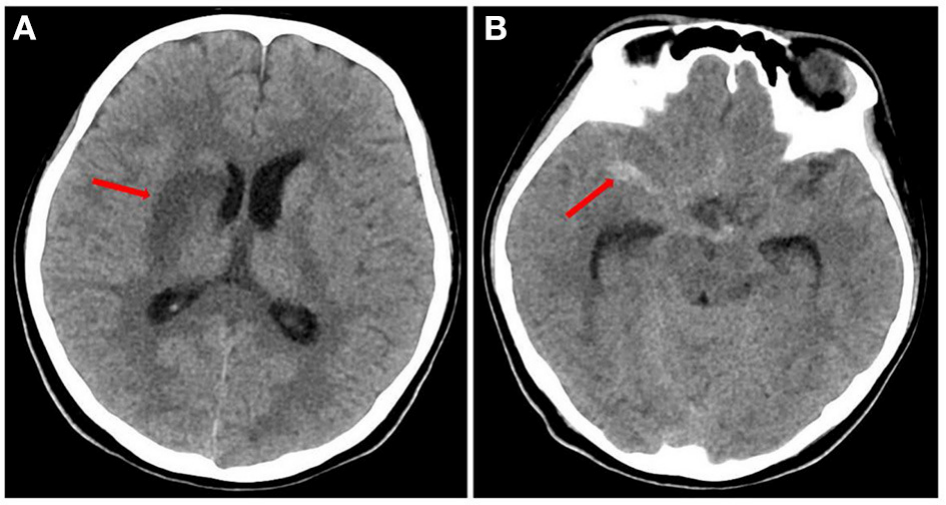
Head CT of the patient **(A)** the red arrow indicates infarction of the right basal ganglia. **(B)** The red arrow indicates subarachnoid hemorrhage.

**Figure 2:**
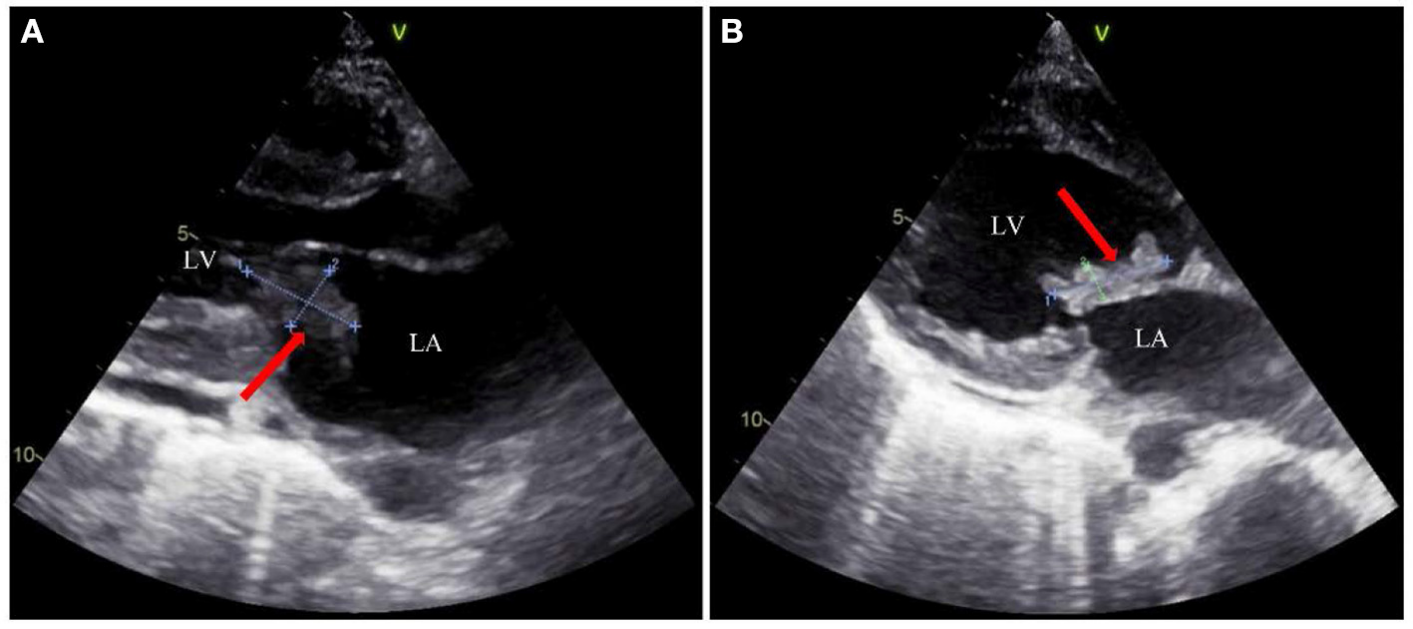
Transthoracic echocardiography examination of the heart in the patient. **(A)** THE red arrow indicates the vegetation (2.3 × 1.3 cm) on the mitral valve. **(B)** The red arrow indicates another position of the vegetation (2.3 × 0.7 cm) on the mitral valve. LA: left atrium; LV: left ventricle.

**Figure 3:**
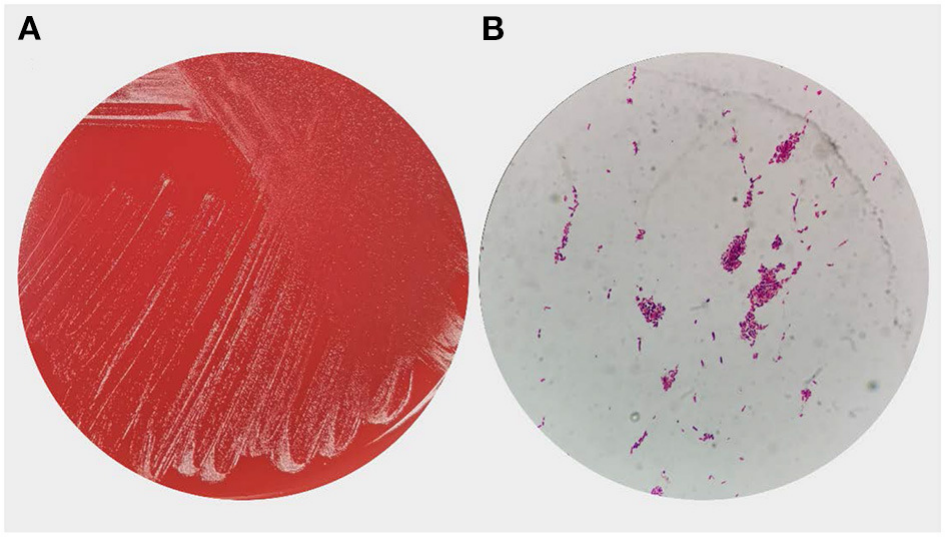
Overview of *Abiotrophia defectiva* of growth morphology **(A)** Colony growth of *A. defectiva* on blood agar. **(B)** Smear showing gram-positive cocci in chains.

A head CT scan urgently performed revealed a cerebral infarction in the right basal ganglia and a subarachnoid hemorrhage (Figure 1). A transthoracic echocardiogram (TTE) revealed vegetation on the mitral leaflets measuring 2.3 × 1.3 cm (Figure 2). Before administering ceftriaxone to the patient, three blood cultures (aerobic and anaerobic bottles from peripheral blood once every half an hour) were obtained. After 9.5 h, the blood culture was positive for facultative anaerobic Gram-positive streptococcus, which was immediately identified as *A. defectiva* (Figure 3) with the Vitek MS using library v3.2 (bioMérieux, Marcy-l'Étoile, France) and the percent of identity is 99.9%. According to antimicrobial susceptibility testing (Supplementary Figure 1), the bacteria were susceptible to vancomycin, clindamycin, and linezolid but resistant to penicillin, ceftriaxone, and cefepime. All three blood cultures tested positive for *A. defectiva*. The girl met two of the modified Duke diagnostic criteria for IE. Based on the susceptibility test results, the antibiotic therapy was immediately changed to vancomycin, 3 days later, the girl's temperature turned normal. The girl was transferred to a higher-level hospital for cardiovascular surgery on the tenth hospital day. Three weeks later, she underwent mitral valve replacement surgery. Upon discharge, two months after the onset of symptoms, her left lower limb muscle strength was grade V, while the left upper limb muscle showed a strength of grade IV.

## Discussion

*A. defectiva* is a facultative anaerobic Gram-positive coccus that was first reported in a case of IE in 1961 (1). *A. defectiva* normally afflicts the respiratory, urogenital, and gastrointestinal tracts (2). The IE caused by *A. defectiva* usually occurs in patients with congenital heart disease or a history of previous cardiac surgery (3). Moreover, oral hygiene, and a dental procedure could also be important causes of *A. defectiva* related IE (4, 5). In the pertinent case, the girl had a history of an orthodontics procedure, which could have contributed to IE. Compared to other pathogens leading to IE, *A. defectiva* is more likely to form valvular vegetations and lead to cardiogenic emboli (6, 7). Literature suggests that due to the production of a considerable amount exopolysaccharides, the organism has a higher affinity for the endocardium and the ability to bind with fibronectin in the extracellular matrix, further contributes to their virulence (8, 9). Colonization and infection of corneas, joints and heart valves may indicate a significant tendency of the bacterium to colonize vascular free collagen tissue (10). Besides, *A. defectiva* endocarditis has an indolent course and is often culture-negative, contributing to a delayed diagnosis, with high mortality, and complication rates (6). The NVS are estimated to cause approximately 5%−6% of all cases of IE (11), the mortality rate of NVS IE is 9.2% (12). In the patient in the pertinent case, the disease course continued for 6 weeks. Unfortunately, the IE was complicated by heart failure, a brain infarction, and subarachnoid hemorrhage when diagnosed.

The patient, in this case, the young girl, was admitted to the NICU with symptoms associated with brain infarction, hence, urgent brain imaging was essential to confirm the stroke diagnosis and guide the therapy strategies. Moreover, the etiology of brain infarction determines the subsequent prevention strategies. Stroke, cardioembolic causes, and arterio-pathic causes are the most frequent etiologies in childhood (defined as 29 days to 18 years of age). The reasons for arterial ischemic stroke include congenital or acquired heart disease, non-atherosclerotic arteriopathies (arterial dissection, focal cerebral arteriopathy, and Moyamoya), and sickle cell diseases (13).

For the diagnosis of IE, an echocardiogram and laboratory testing (especially blood cultures) would be needed. In the current case, timely blood cultures and bacterial mass spectrometry facilitated the rapid identification of a rare pathogen, like *A. defectiva*. The 16S rRNA sequencing also played an important role in verification of *A. defective* (14). Moreover, Metagenomic Next-Generation Sequencing (mNGS) has been reported in the clinical diagnosis of IE (15). Compared to conventional cultures, the mNGS has been found to be more sensitive and particularly suitable for rare or culture-negative pathogens (16). Recently, Du reported a case of IE caused by *A. defectiva*, with the diagnosis being assisted by mNGS (17).

The administration of proper antibiotics is the foundation of the IE treatment. According to the 2015 European Society of Cardiology (ESC) guidelines, penicillin G, ceftriaxone, or vancomycin need to be used for 6 weeks, combined with an aminoglycoside for at least 2 weeks (18). The American Heart Association guidelines recommend the administration of a combination regimen that includes ampicillin (12 g/d in divided doses) or penicillin (18–30 million U/D in divided doses or by continuous infusion) plus gentamicin (3 mg/kg/day in 2–3 divided doses) with infectious diseases consultation to determine the duration of the therapy (19). Almost all susceptibility studies have reported that *A. defectiva* was susceptible to vancomycin. Although *A. defectiva* susceptibility to ceftriaxone was 92%–100% (20), in this case study, the bacteria were resistant to ceftriaxone. In IE caused by *A. defectiva*, approximately 27% of patients need prosthetic valve replacement (21). Due to the large mitral valve vegetations (>1 cm) and severe embolic events, the pertinent patient had to undergo surgical mitral valve replacement.

## Conclusion

This case study indicated that *A. defectiva* is an important cause of IE, which could lead to severe complications. Thus, timely application of blood cultures and rapid identification of *A. defectiva* would enable the proper prescription of antibiotics. Moreover, effective anti-infective medications and surgical interventions need to be combined to avoid and/or manage serious complications.

## Data availability statement

The original contributions presented in the study are included in the article/Supplementary material, further inquiries can be directed to the corresponding authors.

## Ethics statement

Written informed consent was obtained from the individual(s), and minor(s)' legal guardian/next of kin, for the publication of any potentially identifiable images or data included in this article. Written informed consent was obtained from the participant/patient(s) for the publication of this case report.

## Author contributions

MY: diagnosis and treatment of the patient and conception and creation of the manuscript. YL: conceptualization and original draft preparation. XP: writing. JW: microbial identification. BH: clinical consultation. YH: reviewing. JL: supervision and reviewing. All authors contributed to the article and approved the submitted version.

## Supplementary material

Supplementary Figure 1 Antimicrobial susceptibility test results of *Abiotrophia defectiva* according to CLSI M45 criterion. MIC, Minimum Inhibitory Concentration; KB, Kirby-Bauer Test; I, Intermediate; R, Resistant; S, Susceptible.
