# Supplementary figures and images for: Abiotrophia defectiva causing infective endocarditis with brain infarction and subarachnoid hemorrhage: a case report
Source: Front Med (Lausanne). 2023 May 3;10:1117474. doi: 10.3389/fmed.2023.1117474 (PMC10188988; doi:10.3389/fmed.2023.1117474)

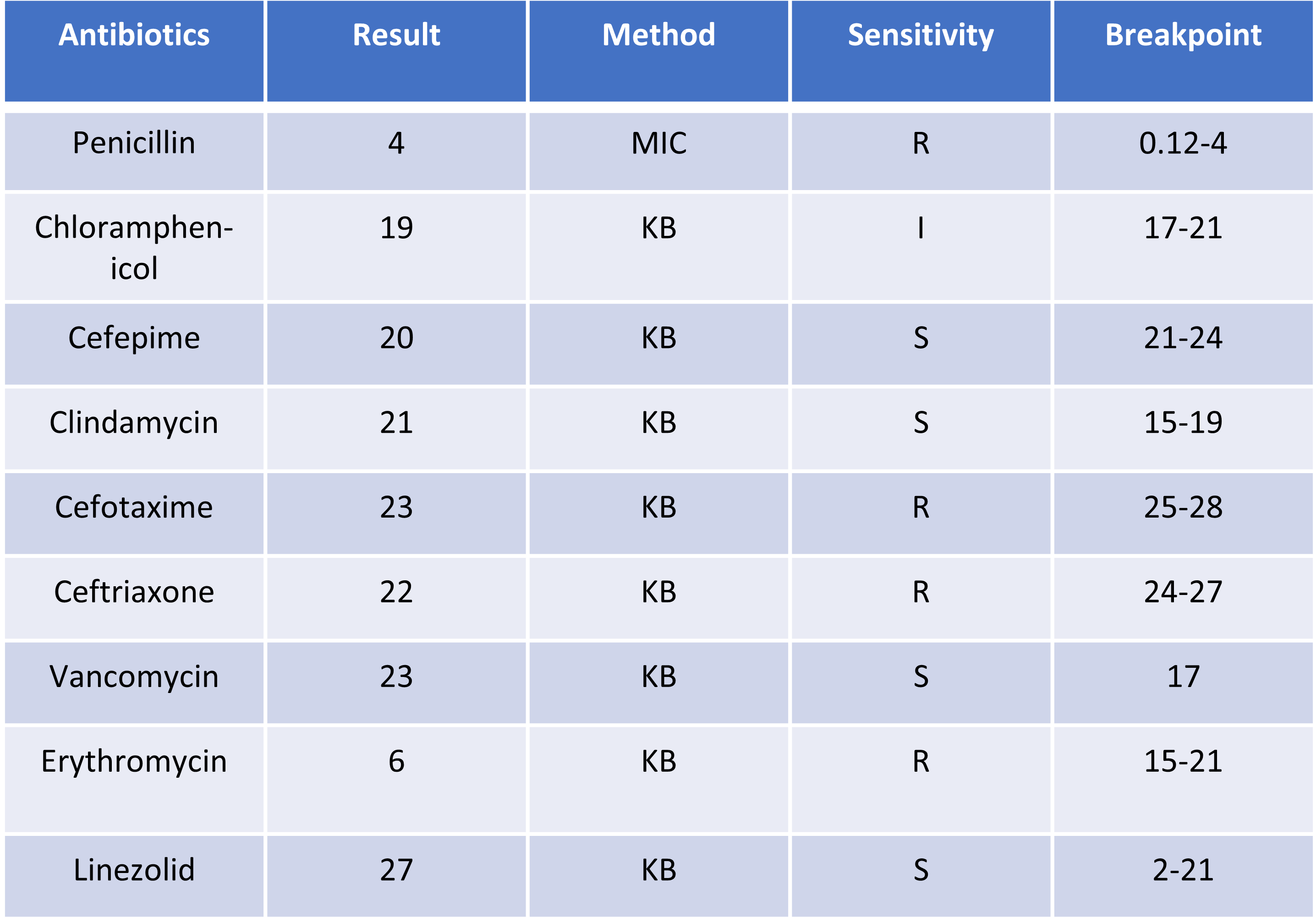

Supplement: Supplementary Figure 1 — Antimicrobial susceptibility test results of Abiotrophia defectiva according to CLSI M45 criterion. MIC, Minimum Inhibitory Concentration; KB, Kirby-Bauer Test; I, Intermediate; R, Resistant; S, Susceptible. [file Image_1.TIF]
